# Effect of clarification on physicochemical properties and nutrient retention of pressed and blended cashew apple juice

**DOI:** 10.1002/fsn3.3222

**Published:** 2023-01-11

**Authors:** Angela Aluko, Edna Makule, Neema Kassim

**Affiliations:** ^1^ Department of Food Biotechnology and Nutritional Sciences Nelson Mandela African Institution of Science and Technology Arusha Tanzania; ^2^ Department of Food Science and Technology Mbeya University of Science and Technology Mbeya Tanzania

**Keywords:** cashew apple juice, clarification, gelatine, pressing and blending, tannin

## Abstract

The cashew apple is a nutritious pseudofruit of the cashew tree, after the nut. It is highly perishable and has an astringent taste, which hinder its utilization in the food sector. This study was designed to optimize clarification of cashew apple juice (CAJ) using gelatine, and assess the effect of clarification on physicochemical properties (titratable acidity, total soluble solids, and pH), nutrient retention, and sensory quality of pressed and blended CAJ. CAJ was treated with gelatine concentrations (0, 0.025, 0.05, 0.1, 0.2, 0.3, and 0.4 g/L) at room temperature (24–26°C) for 1, 2, 4, 6, 8, 10, and 12 h. Both clarified and unclarified juice were analyzed for tannin, total phenol, β‐carotene, vitamin C, sugar, minerals, antioxidant activity, and physicochemical and sensory qualities. The results showed that tannin in pressed CAJ was optimally reduced from 217.6 mg/100 ml TAE to 24.6 mg/100 ml TAE, and from 258.0 mg/100 ml TAE to 55.0 mg/100 ml TAE in blended CAJ, using 0.2 g of gelatine in a liter of juice, for 2 h at room temperature. Both CAJ with and without clarification showed no significant difference in pH, total soluble solids, and titratable acidity (*p* < .05). However, blended CAJ had higher contents of total phenol, tannin, β‐carotene, vitamin C, sugar, minerals, and antioxidant activity (*p* < .05). The use of a low concentration (0.2 g/L) of gelatine in a liter of either blended or pressed CAJ yielded sweet and less astringent CAJ with high‐intensity yellow color. The technologies performed well at room temperature and therefore provide the basis for potential business investment.

## Graphical abstract

Clarification on quality of cashew apple juice blending and pressing techniques for cashew apple juice extraction.

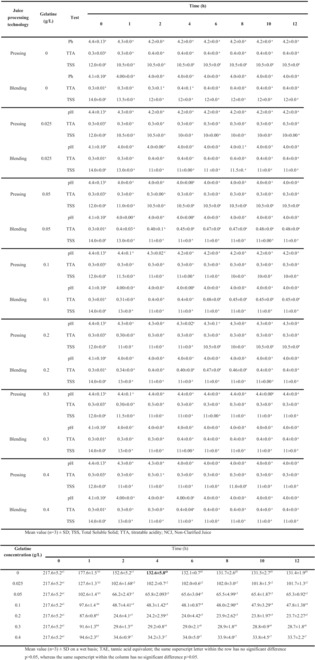

## 1 INTRODUCTION

The cashew apple is a tropical fruit rich in vitamin C, averaging from 200 to 269 mg/100 ml of juice (Prasertsri & Leelayuwat, 2017), and five times that of citrus juice and 10 times that of pineapple juice (Akinwale, 2000; Azam‐Ali & Judge, 2001; Naka et al., 2016). It also contains other vitamins, such as thiamine, niacin, and riboflavin, and precursors of vitamin A. In addition, it contains copper, zinc, sodium, potassium, calcium, iron, phosphorus, and magnesium (Lowor & Agyente‐Badu, 2009), sulfur, silicon, chlorine, aluminum, and bromine (Marc et al., 2011). Furthermore, cashew apple juice can be used for medical purposes to treat sore throat and chronic dysentery (Aderiye et al., 2014; Bhakyaraj & Singaravadive, 2012). Besides, the fruit contains 35% polyphenol components and 3% of unknown oily compounds, which are considered the cause of juice's astringency (Adegunwa et al., 2020; Azevedo & Rodrigues, 2000; Jayalekshmy & John, 2004).

Due to its delicate skin that increases susceptibility to bruises and insect damage, the fruit is highly perishable limiting its transportation and storability (Preethi et al., 2019; Sivakumar et al., 2011). Moreover, the fruit contains high tannin content ranging from 1081.99 to 2561.61 mg/L (Naka et al., 2015), which is the main cause of its astringent taste. Both the perishability and astringent taste have hindered its marketability and utilization (Campos et al., 2002; Naka et al., 2016). Although the processing of cashew apples into various products such as concentrate, juice, jam, wine, jelly, energy drinks, and dried flakes has been used to navigate through perishability challenges, these processes may require clarification to reduce astringency. Especially in cashew apple juice processing, the use of clarifying agents to remove tannins (polyphenols) has been used to improve its palatability (Awe, 2018; Naka et al., 2016).

A clarifier, also known as fining agent, improves the clarity of the juice by forming an adsorbent, enzymatic, or ionic bond with suspended particles, allowing them to precipitate out of the juice more easily and quickly (Berry, 2000). Unlike filtration, which can only remove particulates like fruit bits, fining effectively removes soluble substances such as polymerized tannins, coloring phenols, and proteins (Awe, 2018). Clarifying agents such as sago starch, gelatine, polyvinyl pyrolidone, starch, rice gruel (Campos et al., 2002; Cormier, 2008; Jayalekshmy & John, 2004), and tannase enzyme (Kundu et al., 2022) have been experimented for their usefulness in removing tannins from cashew apple juice.

Although a majority of these clarifying agents have shown promising results, gelatine has shown to be relatively more effective in pomegranate (Vardin & Fenercioǧlu, 2003) and cashew apple (Prommajak et al., 2018) juice clarification. In comparison with other protein‐based clarifying agents such as albumin and casein, gelatine is more effective; this is due to the fact that it has a positive charge at a pH level lower than its isoelectric point (pI) (Prommajak et al., 2019). When compared to polyvinylpolypyrrolidone (PVPP) which eliminates all types of polyphenols, gelatine eliminates only the postbottling haze‐forming polyphenols, hence, frequently used as a clarifying agent in juice processing (Benitez & Lozano, 2007).

Despite all these promising technologies and the fact that cashew apples (CA) are an excellent source of vitamins and minerals with various health and nutritional benefits, only about 10% of the cashew apples are utilized as fruits for domestic consumption in Africa. The rest are left to rot on farmers' fields (Akyereko et al., 2022), or only in some cases, a small portion is fed to animals. Tanzania, for instance, recorded approximately 2,327,000 tons of cashew apple production in the year 2019/2020 (Cashewnut Board of Tanzania, 2022), of which more than 80% was partly left to rot on the farms or fed to animals. The production is projected to increase to approximately 7 million tons of cashew apples by the year 2025/2026 due to the underway interventions of spreading cashew production in other parts of the country (Dimoso, Aluko, et al., 2020).

Although CAJ clarification technologies have been reported (Ukonze et al., 2018), their application in low‐resource settings remains challenging, creating a customization gap. Therefore, this study was designed to optimize clarification of CAJ in low‐resource settings using gelatine, and assess the effect of clarification on physicochemical properties, nutrient retention, and sensory qualities of pressed and blended CAJ.

## 2 MATERIALS AND METHODS

### 2.1 Study site and collection of cashew apple

Cashew apples (CA) were obtained from cashew farms owned by Tanzania Agriculture Research Institute (TARI)—Naliendele, located at Nachingwea (10°19′46″S, 38°46′46″E; 442 masl), Lindi Region. The fully matured and ripe apples of Brazilian dwarf variety were handpicked randomly from the cashew trees early in the morning at 6:30 am. The variety was chosen because it has minimum tannin (151.73–376.44 mg/L) and high nutrient content, particularly vitamin C (294.70–341.17 mg/100 ml) (Dimoso, Aluko, et al., 2020; Msoka et al., 2017). The handpicked CAs were carefully packed in plastic crates to three quarters to allow ventilation and avoid pressing of fruits in the lower crates upon stacking. Crates were placed onto an enclosed air‐conditioned vehicle at about 18°C, stacked on each other, and transported to the Nelson Mandela African Institution of Science and Technology (NM‐AIST) within 20 h, inclusive of harvest and transportation. Upon arrival, apples were immediately hydrocooled for about 10 min using tap water and stored at −20°C prior to processing of juice and analysis.

### 2.2 Juice preparation and clarification

Intact cashew apples were detached from the nut, washed with potable water, air‐dried, and then weighed. They were then cut into small pieces, pressed using a manual pressing machine to obtain pressed CAJ (PCAJ), or blended with a kitchen blender for about 3 min to obtain blended CAJ (BCAJ). The juices were then filtered through a muslin cloth. Juice clarification was done using gelatine according to the methods adopted from Remyamol (2006); Talasila et al. (2012), with some modifications. Ten percent (10%) of gelatine solution was prepared by dissolving 10 g of gelatine in 100 ml of hot water (100°C).

Then, the mixture was stirred thoroughly to dissolve. The dissolved gelatine in 0.25, 0.5, 1, 2, 3, and 4 ml was each added into 1 L of BCAJ or PCAJ to make a concentration of 0.025, 0.05, 0.1, 0.2, 0.3, and 0.4 g/L, respectively. The mixture was stirred well and left to clarify at ambient temperature (24–26°C) for 1, 2, 4, 6, 8, 10, and 12 h. After each of the specified clarification times, the juice was decanted into clean vessels. The clarified cashew apple juice alongside unclarified cashew apple juice as controls were analyzed for tannin and physicochemical characteristics. The optimally clarified juice in terms of low tannin content and shorter clarification time was selected for further analysis of minerals, vitamin C, β‐carotene, sugar, total phenol, sensory quality, and antioxidant activity. However, for sensory quality, juices were pasteurized at 75°C for 5 min to eliminate microbes. Some cooled juices were added with preservative citric acid (0.01%) and sodium benzoate (0.01%).

### 2.3 Determination of pH, total titratable acidity, and total soluble solids

pH was determined using a pH meter (Orion star A214) calibrated at three buffer solutions of pH 4, 7, and 10. Total titratable acidity (TTA) as a percent of citric acid was determined by placing 6 ml of the CAJ in a beaker, diluted with 50 ml of distilled water, and titrated against 0.1 M sodium hydroxide (LOBA Chemie) solution until the endpoint at pH 8.2 (Adou et al., 2012; Sadler & Murphy, 2010). The formula used for calculating total titratable acidity was as follows:

$$\%\text{acid}\;\left(\frac{\mathrm{wt}}{\mathrm{wt}}\right)=\frac{N\times V\times \text{Eqwt}}{W\times 1000}\times 100$$

where *N* = normality of titrant, usually NaOH (mEq/ml); *V* = volume of titrant (ml); Eq. wt. = equivalent weight of predominant acid (mg/mEq); *W* = mass of sample (g); 1000 = factor relating mg to grams (mg/g) (1/10 = 100/1000).

Total soluble solids (TSS) were determined using a portable hand‐held refractometer (GriffChem), equipped with a digital display and expressed as °Brix (Adou et al., 2012).

### 2.4 Determination of total phenolic content

The total phenolic content of CAJ was determined in terms of gallic acid equivalents (GAE) using the Folin‐Ciocalteu reagent (Singleton & Rossi, 1965) with slight modification. One milliliter of CAJ was diluted with distilled water to 10 ml. An aliquot (0.5 ml) from the diluted sample was mixed with 2.4 ml of distilled water, 2 ml of 2% Na_2_CO_3_ (LOBA Chemie), and 0.1 ml Folin–Ciocalteu (LOBA Chemie). The mixture was incubated at room temperature in a dark place for 60 min (Mahdavi et al., 2011). The absorbance of the sample was measured at 750 nm using an ultraviolet–visible (UV–Vis) spectrophotometer multimode reader, SYNERGY/HTX (BioTek). Total phenolic content was then calculated based on the standard curve of gallic acid (5, 15, 20, 25, and 50 mg/100 ml) with equation *y* = 0.0665*x* + 0.2767 and *R* ^2^ = .9829 and expressed as mg/100 ml of gallic acid equivalent (GAE).

### 2.5 Determination of tannins content

Tannin contents were determined by the Folin–Ciocalteu reagent in terms of tannic acid equivalent (TAE) (Chandran & Indira, 2016). About 0.1 ml of CAJ was added to a volumetric flask containing 7.5 ml of distilled water and 0.5 ml of Folin–Ciocalteu phenol reagent (LOBA Chemie), 1 ml of 35% sodium carbonate solution (LOBA Chemie), and diluted to 10 ml with distilled water. The mixture was shaken well and kept at room temperature for 30 min. A set of reference standard solutions of tannic acid (20, 40, 60, 80, and 100 mg/100 ml) with equation *y* = 0.0164*x* + 0.1848 and *R* ^2^ = .9512 were prepared in the same manner as described on the CAJ sample. Absorbance for test and standard solutions were measured against the blank at 700 nm with a UV/visible spectrophotometer, multimode reader, SYNERGY/HTX (BioTek). The estimation of the tannin content was carried out in triplicate. The tannin content was expressed in terms of mg of tannic acid equivalents/100 ml of the sample.

### 2.6 Determination of vitamin C

Vitamin C content was determined as described by Desai and Desai (2019) and Dimoso, Makule, and Kassim (2020). Briefly, 5 ml of the sample was mixed with 25 ml of metaphosphoric acid–acetic acid solution (LOBA Chemie) and centrifuged (Eppendorf 5810) at 4000 rpm for 15 min. Four milliliters (4 ml) of the extract was treated with 0.23 ml of bromine water (3%) (LOBA Chemie), followed by 0.13 ml of 10% thiourea solution (LOBA Chemie), and then 1 ml of 2, 4 dinitrophenylhydrazine solution (LOBA Chemie) was added. The mixture was kept in a thermostatic water bath at 37°C for 3 h, cooled for 30 min, and then treated with 6 ml of chilled (4°C) 85% sulfuric acid (LOBA Chemie). The absorbance of the resulting red‐colored solution was measured spectrophotometrically at 521 nm, with a UV/visible spectrophotometer (UV–Vis, multimode reader, SYNERGY/HTX [BioTek]). The total ascorbic acid content was estimated based on the standard curve of ascorbic acid (10, 20, 30, 40, and 50 mg/100 ml), with equation *y* = 0.0098*x* + 0.0857 and *R* ^2^ = .9872, and the result was expressed as mg/100 ml.

### 2.7 Determination of β‐carotene

Beta carotene was determined as described by Perez‐Lopez (2010) and AOAC (1980) with slight modification. Five ml of CAJ was placed into a 50‐ml falcon tube. Then, 30 ml of extraction solvent; hexane:acetone:ethanol (2:1:1, v/v) (LOBA Chemie) was added, and the mixture was centrifuged (Eppendorf 5810) at 4000 rpm for 10 min. The supernatant was transferred into a separating funnel, and 50 ml of 10% sodium chloride (LOBA Chemie) was added to remove residual acetone. The upper phase was recovered, dried over anhydrous sodium sulfate (LOBA Chemie), and the absorbance was determined at 450 nm (UV/Vis spectrophotometer, multimode reader, SYNERGY/HTX [BioTek]). The concentration of β‐carotene in the test sample was estimated based on the standard curve (20, 40, 60, 80, and 100 mg/100 ml), and equation *y* = 0.0449*x* + 0.1807 and *R* ^2^ = .986. The content of β‐carotene was expressed as mg/100 ml.

### 2.8 Determination of mineral contents

The mineral contents of CAJ were determined by energy‐dispersive X‐ray fluorescence spectrometer (EDXRF‐Rigaku Nex CG) as described by Yamada (2014). Briefly, 4 ml of CAJ sample was poured directly into a sample cell, covered, and fixed to the machine. The flow rate of the helium gas was set at 0.66 L/min. Contents of potassium, calcium, zinc, iron, magnesium, and phosphorus were determined and expressed as mg/100 ml.

### 2.9 Determination of sugar content

Ten milliliters of cashew apple juice was centrifuged at 7500 rpm for 5 min. One milliliter of the supernatant was diluted 100 folds with distilled water, and then the total sugar content was determined by the phenol sulfuric acid method (Dubois et al., 1956). Briefly, 2 ml of the diluted sample/standard was pipetted to a 15‐ml test tube; then 50 μl of 80% w/v phenol (LOBA Chemie) was added to both standards and samples. Five milliliters of concentrated H_2_SO_4_ (LOBA Chemie) was added to both sample and standard after 15 min. The sample and standard mixtures were shaken gently and allowed to stand for 10 min at room temperature, and then both samples and standard were placed in a water bath at 27°C for 15 min. Two hundred microliter of samples and standards were placed in microplates wells, and the absorbance of the mixture was measured at 485 nm using multimode reader, SYNERGY/HTX (BioTek), and the concentration in mg/100 ml of the sample was estimated using a calibration curve of the standard glucose (20, 40, 60, 80, and 100 mg/100 ml) with equation *y* = 0.0086*x* + 0.1164 and *R* ^2^ = .9892.

### 2.10 Antioxidant activity by DPPH assay

A radical solution was prepared by dissolving 2.4 mg DPPH in 100 ml methanol. A test solution (5 μl) of CAJ was added to 3.995 ml of methanolic DPPH. The mixture was shaken vigorously and kept at room temperature for 30 min in the dark. The absorbance of the reaction mixture was measured at 515 nm spectrophotometrically. The absorbance of the DPPH radical without antioxidant (blank) was also measured. All the determinations were performed in triplicate. The capability of CAJ to scavenge the DPPH radical was calculated using the following equation (Rajurkar & Hande, 2011).

$$\text{DPPH Scavenged}\;\left(\%\right)=\frac{\mathrm{AB}-\mathrm{AA}}{\mathrm{AB}}\times 100$$

where AB is the absorbance of blank at *t* = 0 min; AA is the absorbance of the antioxidant at *t* = 30 min. The results were expressed as a percentage of DPPH scavenging activity.

### 2.11 Sensory analysis

A quantitative descriptive sensory analysis of cashew apple juice was conducted as described by Da Silva et al. (2014) and Stone (1992). Twelve trained panelists, seven men and five women aged between 20 and 50 years, scored the juice. The panel was selected as described by Stone and Sidel (1998), Meilgaard et al. (2006), and Da Silva et al. (2014). Training of the panel was done for 10 h using the generic descriptive evaluation method as per Lawless and Heymann (2010). During the training, fresh CA juices to be tested were presented to the panelists to generate terms to describe the difference in sensory properties. Through consensus, the sensory descriptors and definitions were developed for the actual sensory evaluation as illustrated in Table 1. A 9‐point scale was used to rate the differences in sensory attributes. Panelists were asked to refrain from smoking, snacking, or chewing gum 20 min before the sensory evaluation session. During the evaluation session, samples of CA juice were presented at room temperature, served in transparent cups, and each sample was coded with three‐digit random number. To avoid bias, the samples were presented in a random order to the panelists. The panelists were asked to evaluate each juice individually at each testing. Portable water was provided to the panelists to rinse their palate before and between testing samples.

**TABLE 1:** Definition of sensory attributes used in qualitative descriptive sensory analysis

Attribute	Definition	Reference
*Appearance*		
Yellow	CAJ at ideal conditions of consumption (nonoxidized)	Strong: CAJ added by a 5% solution of citric acid followed by refrigerating Weak: CAJ stored at room temperature for 24 h and exposed to oxygen.
Brown	CAJ stored at room temperature and exposed to the action of oxygen	Strong: CAJ stored at room temperature for 24 h and exposed to the action of oxygen. Weak: CAJ added by a 5% solution of citric acid followed by refrigerating
*Aroma*		
—	Like the aroma of fresh cashew apple	Fresh cashew apple
*Taste*		
Astringent	CAJ with a taste that induces the tongue's constriction	Strong: CAJ added 0.5% tannin. Weak: CAJ added 50% of water.
Bitter	CAJ with a taste that is sharp, acrid, and unpleasant	Strong: CAJ added 0.1% of caffeine solution. Weak: CAJ added 50% of water.
Sweet	CAJ with a taste of honey or sugar	Strong: CAJ added 20% sucrose. Weak: CAJ pulp plus 50% water.

### 2.12 Statistical analysis

All analyses were performed in triplicate, and values were presented as average mean values with standard derivations. Data were analyzed using the SPSS statistical software version 21. Statistical comparisons were performed with a one‐way analysis of variance, and *p* values <.05 were regarded as significant. The mean differences were analyzed using the least significant (LSD) test.

## 3 RESULTS AND DISCUSSION

The study assessed the effect of clarification of pressed and blended cashew apple juice on physicochemical properties, tannin removal, nutrient retention, and antioxidant activity using DPPH assay. The results are herein presented.

### 3.1 Physicochemical properties of cashew apple juice

Results showed that pH, TSS, and TTA ranged from 4.0 to 4.4, 10.5 to 14 °Brix, and 0.3 to 0.48, respectively, for both clarified and unclarified PCAJ. For clarified and unclarified blended CAJ, pH, TSS, and TTA ranged from 4.06 to 4.08, 13 to 14°Brix, and 0.31 to 0.33, respectively (Table 2). In both pressed and blended juices, clarification had no significant effect on pH, TSS, or TTA (*p* > .05). Unclarified and clarified pressed and blended CAJ has pH and TSS values similar to the values recommended by the Brazilian Agricultural Research Company; pH (3.5–4.6), TSS (9.8–14° Brix), and TTA 0.3% (Dias‐Souza et al., 2017; Maia et al., 2019). Titratable acidity and pH value were lower, while total soluble solids were higher than those reported by Adou et al. (2012).

**TABLE 2:** Physicochemical properties of clarified and unclarified CAJ

Juice processing technology	Gelatine (g/L)	Test	Time (h)							
			0	1	2	4	6	8	10	12
Pressing	0	pH	4.4 ± 0.13^a^	4.3 ± 0.0^a^	4.2 ± 0.0^a^	4.2 ± 0.0^a^	4.2 ± 0.0^a^	4.2 ± 0.0^a^	4.2 ± 0.0^a^	4.2 ± 0.0^a^
		TTA	0.3 ± 0.03^a^	0.3 ± 0.0^a^	0.4 ± 0.0^a^	0.4 ± 0.0^a^	0.4 ± 0.0^a^	0.4 ± 0.0^a^	0.4 ± 0.0^a^	0.4 ± 0.0^a^
		TSS	12.0 ± 0.0^a^	10.5 ± 0.0^a^	10.5 ± 0.0^a^	10.5 ± 0.0^a^	10.5 ± 0.0^a^	10.5 ± 0.0^a^	10.5 ± 0.0^a^	10.5 ± 0.0^a^
Blending	0	pH	4.1 ± 0.10^a^	4.00 ± 0.0^a^	4.0 ± 0.0^a^	4.0 ± 0.0^a^	4.0 ± 0.0^a^	4.0 ± 0.0^a^	4.0 ± 0.0^a^	4.0 ± 0.0^a^
		TTA	0.3 ± 0.01^a^	0.3 ± 0.0^a^	0.3 ± 0.1^a^	0.4 ± 0.1^a^	0.4 ± 0.0^a^	0.4 ± 0.0^a^	0.4 ± 0.0^a^	0.4 ± 0.0^a^
		TSS	14.0 ± 0.0^a^	13.5 ± 0.0^a^	12 ± 0.0^a^	12 ± 0.0^a^	12 ± 0.0^a^	12 ± 0.0^a^	12 ± 0.0^a^	12 ± 0.0^a^
Pressing	0.025	pH	4.4 ± 0.13^a^	4.3 ± 0.0^a^	4.2 ± 0.0^a^	4.2 ± 0.0^a^	4.2 ± 0.0^a^	4.2 ± 0.0^a^	4.2 ± 0.0^a^	4.2 ± 0.0^a^
		TTA	0.3 ± 0.03^a^	0.3 ± 0.0^a^	0.3 ± 0.0^a^	0.3 ± 0.0^a^	0.3 ± 0.0^a^	0.3 ± 0.0^a^	0.3 ± 0.0^a^	0.3 ± 0.0^a^
		TSS	12.0 ± 0.0^a^	10.5 ± 0.0^a^	10.5 ± 0.0^a^	10 ± 0.0^a^	10 ± 0.00^a^	10 ± 0.0^a^	10 ± 0.0^a^	10 ± 0.00^a^
Blending	0.025	pH	4.1 ± 0.10^a^	4.0 ± 0.0^a^	4.0 ± 0.00^a^	4.0 ± 0.0^a^	4.0 ± 0.0^a^	4.0 ± 0.1^a^	4.0 ± 0.0^a^	4.0 ± 0.0^a^
		TTA	0.3 ± 0.01^a^	0.3 ± 0.0^a^	0.4 ± 0.0^a^	0.4 ± 0.0^a^	0.4 ± 0.0^a^	0.4 ± 0.0^a^	0.4 ± 0.0^a^	0.4 ± 0.0^a^
		TSS	14.0 ± 0.0^a^	13.0 ± 0.0^a^	11 ± 0.0^a^	11 ± 0.00^a^	11 ± 0.0^a^	11.5 ± 0.0^a^	11 ± 0.0^a^	11 ± 0.0^a^
Pressing	0.05	pH	4.4 ± 0.13^a^	4.0 ± 0.0^a^	4.0 ± 0.0^a^	4.0 ± 0.00^a^	4.0 ± 0.0^a^	4.0 ± 0.0^a^	4.0 ± 0.0^a^	4.0 ± 0.0^a^
		TTA	0.3 ± 0.03^a^	0.3 ± 0.0^a^	0.3 ± 0.00^a^	0.3 ± 0.0^a^	0.3 ± 0.0^a^	0.3 ± 0.0^a^	0.3 ± 0.0^a^	0.3 ± 0.0^a^
		TSS	12.0 ± 0.0^a^	11.0 ± 0.0^a^	10.5 ± 0.0^a^	10.5 ± 0.0^a^	10.5 ± 0.0^a^	10.5 ± 0.0^a^	10.5 ± 0.0^a^	10.5 ± 0.0^a^
Blending	0.05	pH	4.1 ± 0.10^a^	4.0 ± 0.00^a^	4.0 ± 0.0^a^	4.0 ± 0.00^a^	4.0 ± 0.0^a^	4.0 ± 0.0^a^	4.0 ± 0.0^a^	4.0 ± 0.0^a^
		TTA	0.3 ± 0.01^a^	0.4 ± 0.03^a^	0.40 ± 0.1^a^	0.45 ± 0.0^a^	0.47 ± 0.0^a^	0.47 ± 0.0^a^	0.48 ± 0.0^a^	0.48 ± 0.0^a^
		TSS	14.0 ± 0.0^a^	13.0 ± 0.0^a^	11 ± 0.0^a^	11 ± 0.0^a^	11 ± 0.0^a^	11 ± 0.0^a^	11 ± 0.00^a^	11 ± 0.0^a^
Pressing	0.1	pH	4.4 ± 0.13^a^	4.4 ± 0.1^a^	4.3 ± 0.02^a^	4.2 ± 0.0^a^	4.2 ± 0.0^a^	4.2 ± 0.0^a^	4.2 ± 0.0^a^	4.2 ± 0.0^a^
		TTA	0.3 ± 0.03^a^	0.3 ± 0.0^a^	0.3 ± 0.0^a^	0.3 ± 0.0^a^	0.3 ± 0.0^a^	0.3 ± 0.0^a^	0.3 ± 0.0^a^	0.3 ± 0.0^a^
		TSS	12.0 ± 0.0^a^	11.5 ± 0.0^a^	11 ± 0.0^a^	11 ± 0.00^a^	11 ± 0.0^a^	10 ± 0.0^a^	10 ± 0.0^a^	10 ± 0.0^a^
Blending	0.1	pH	4.1 ± 0.10^a^	4.00 ± 0.0^a^	4.0 ± 0.0^a^	4.0 ± 0.00^a^	4.0 ± 0.0^a^	4.0 ± 0.0^a^	4.0 ± 0.0^a^	4.0 ± 0.0^a^
		TTA	0.3 ± 0.01^a^	0.31 ± 0.0^a^	0.4 ± 0.0^a^	0.4 ± 0.0^a^	0.48 ± 0.0^a^	0.45 ± 0.0^a^	0.45 ± 0.0^a^	0.45 ± 0.0^a^
		TSS	14.0 ± 0.0^a^	13 ± 0.0^a^	11 ± 0.0^a^	11 ± 0.0^a^	11 ± 0.0^a^	11 ± 0.0^a^	11 ± 0.0^a^	11 ± 0.0^a^
Pressing	0.2	pH	4.4 ± 0.13^a^	4.3 ± 0.0^a^	4.3 ± 0.0^a^	4.3 ± 0.02^a^	4.3 ± 0.1^a^	4.3 ± 0.0^a^	4.3 ± 0.0^a^	4.3 ± 0.0^a^
		TTA	0.3 ± 0.03^a^	0.30 ± 0.0^a^	0.3 ± 0.0^a^	0.3 ± 0.0^a^	0.3 ± 0.0^a^	0.3 ± 0.0^a^	0.3 ± 0.0^a^	0.3 ± 0.0^a^
		TSS	12.0 ± 0.0^a^	11 ± 0.0^a^	11 ± 0.0^a^	11 ± 0.0^a^	10.5 ± 0.0^a^	10 ± 0.0^a^	10.5 ± 0.0^a^	10.5 ± 0.0^a^
Blending	0.2	pH	4.1 ± 0.10^a^	4.0 ± 0.0^a^	4.0 ± 0.0^a^	4.0 ± 0.0^a^	4.0 ± 0.0^a^	4.0 ± 0.0^a^	4.0 ± 0.0^a^	4.0 ± 0.0^a^
		TTA	0.3 ± 0.01^a^	0.34 ± 0.0^a^	0.4 ± 0.0^a^	0.40 ± 0.0^a^	0.47 ± 0.0^a^	0.46 ± 0.0^a^	0.4 ± 0.0^a^	0.4 ± 0.0^a^
		TSS	14.0 ± 0.0^a^	13 ± 0.0^a^	11 ± 0.0^a^	11 ± 0.0^a^	11 ± 0.0^a^	11 ± 0.0^a^	11 ± 0.00^a^	11 ± 0.0^a^
Pressing	0.3	pH	4.4 ± 0.13^a^	4.4 ± 0.1^a^	4.4 ± 0.0^a^	4.4 ± 0.0^a^	4.4 ± 0.0^a^	4.4 ± 0.0^a^	4.4 ± 0.00^a^	4.4 ± 0.0^a^
		TTA	0.3 ± 0.03^a^	0.30 ± 0.0^a^	0.3 ± 0.0^a^	0.3 ± 0.0^a^	0.3 ± 0.0^a^	0.3 ± 0.0^a^	0.3 ± 0.0^a^	0.3 ± 0.0^a^
		TSS	12.0 ± 0.0^a^	11.5 ± 0.0^a^	11 ± 0.0^a^	11 ± 0.0^a^	11 ± 0.00^a^	11 ± 0.0^a^	11 ± 0.0^a^	11 ± 0.0^a^
Blending	0.3	pH	4.1 ± 0.10^a^	4.0 ± 0.0^a^	4.0 ± 0.0^a^	4.0 ± 0.0^a^	4.0 ± 0.0^a^	4.0 ± 0.0^a^	4.0 ± 0.0^a^	4.0 ± 0.0^a^
		TTA	0.3 ± 0.01^a^	0.3 ± 0.0^a^	0.3 ± 0.0^a^	0.4 ± 0.0^a^	0.4 ± 0.0^a^	0.4 ± 0.0^a^	0.4 ± 0.0^a^	0.4 ± 0.0^a^
		TSS	14.0 ± 0.0^a^	13 ± 0.0^a^	11 ± 0.0^a^	11 ± 0.00^a^	11 ± 0.0^a^	11 ± 0.0^a^	11 ± 0.0^a^	11 ± 0.0^a^
Pressing	0.4	pH	4.4 ± 0.13^a^	4.3 ± 0.0^a^	4.3 ± 0.0^a^	4.0 ± 0.0^a^	4.0 ± 0.0^a^	4.0 ± 0.0^a^	4.0 ± 0.0^a^	4.0 ± 0.0^a^
		TTA	0.3 ± 0.03^a^	0.3 ± 0.0^a^	0.3 ± 0.1^a^	0.3 ± 0.0^a^	0.3 ± 0.0^a^	0.3 ± 0.0^a^	0.3 ± 0.0^a^	0.3 ± 0.0^a^
		TSS	12.0 ± 0.0^a^	11 ± 0.0^a^	11 ± 0.0^a^	11 ± 0.0^a^	11 ± 0.0^a^	11.0 ± 0.0^a^	11 ± 0.0^a^	11 ± 0.0^a^
Blending	0.4	pH	4.1 ± 0.10^a^	4.00 ± 0.0^a^	4.0 ± 0.0^a^	4.00 ± 0.0^a^	4.0 ± 0.0^a^	4.0 ± 0.0^a^	4.0 ± 0.0^a^	4.0 ± 0.0^a^
		TTA	0.3 ± 0.01^a^	0.3 ± 0.0^a^	0.3 ± 0.0^a^	0.4 ± 0.04^a^	0.4 ± 0.0^a^	0.4 ± 0.0^a^	0.4 ± 0.0^a^	0.4 ± 0.0^a^
		TSS	14.0 ± 0.0^a^	13 ± 0.0^a^	11 ± 0.0^a^	11 ± 0.0^a^	11 ± 0.0^a^	11 ± 0.0^a^	11 ± 0.0^a^	11 ± 0.0^a^

*Note*: Mean value (*n* = 3) ± SD.

Abbreviations: NCJ, nonclarified juice; TSS, total soluble solid; TTA, titratable acidity.

^a^ Significance was at *p* value less than or equal to .05.

Moreover, Msoka et al. (2017) reported similar pH and TTA, but the results of total soluble solids were higher (14.56–20.36) than those of the current study. The physicochemical results in this study imply that the cashew apples used were at optimal maturity (Msoka et al., 2017). This is due to the fact that at optimum maturity the acidity is low (Msoka et al., 2017).

### 3.2 Tannin content of cashew apple juice

Tannin contents were measured in both blended and pressed, clarified and unclarified juice (Tables 3 and 4). The unclarified blended juice had higher tannin content (258 mg/100 ml TAE) compared to unclarified pressed juice (217.6 mg/100 ml TAE) (*p* < .05). The high content of tannin in the unclarified blended CAJ is caused by the high amount of tannin in the skin of the fruit (Kyraleou et al., 2019) as blending involves crushing the whole fruit including the skin. On the other hand, pressed juice involves the squeezing of the fruit to obtain only the juice that does not contain the skin. Tannins content found in unclarified blended CAJ in this study was relatively higher, compared to those of 1081.99–2561.61 mg/L GAE, reported by Naka et al. (2015). Generally, tannin contents found in pressed and blended CAJ, in this study, were higher compared to those of other fruits such as banana (8.38–9.79 mg/100 g) (Kyamuhangire et al., 2006) and apple (8.5 mg/100 g) (Obion et al., 2007).

**TABLE 3:** Reduction of tannin from pressed CAJ using gelatine (mg/100 mL TAE)

Gelatine concentration (g/L)	Time (h)							
	0	1	2	4	6	8	10	12
0	217.6 ± 5.2^a1^	177.6 ± 1.5^b1^	152.6 ± 5.2^c1^	**132.6 ± 5.8** ^**d1**^	132.1 ± 0.7^d1^	131.7 ± 2.6^d1^	131.5 ± 2.7^d1^	131.4 ± 1.9^d1^
0.025	217.6 ± 5.2^a1^	127.6 ± 1.3^b2^	102.6 ± 1.68^c2^	102.2 ± 0.7^c2^	102.0 ± 0.6^c2^	102.0 ± 3.0^c2^	101.8 ± 1.5^c2^	101.7 ± 1.3^c2^
0.05	217.6 ± 5.2^a1^	102.6 ± 1.4^b3^	66.2 ± 2.43^c3^	65.8 ± 2.093^c3^	65.6 ± 3.04^c3^	65.5 ± 4.99^c3^	65.4 ± 1.87^c3^	65.3 ± 0.92^c3^
0.1	217.6 ± 5.2^a1^	97.6 ± 1.4^b4^	48.7 ± 4.41^c4^	48.3 ± 1.42^c4^	48.1 ± 0.87^c4^	48.0 ± 2.90^c4^	47.9 ± 3.29^c4^	47.8 ± 1.38^c4^
0.2	217.6 ± 5.2^a1^	87.6 ± 0.8^b5^	24.6 ± 4.1^c5^	24.2 ± 2.59^c5^	24.0 ± 4.42^c5^	23.9 ± 2.62^c5^	23.8 ± 1.97^c5^	23.7 ± 2.27^c5^
0.3	217.6 ± 5.2^a1^	91.6 ± 1.3^b6^	29.6 ± 1.3^c6^	29.2 ± 0.8^c6^	29.0 ± 2.1^c6^	28.9 ± 1.8^c6^	28.8 ± 0.9^c6^	28.7 ± 1.8^c6^
0.4	217.6 ± 5.2^a1^	94.6 ± 2.3^b7^	34.6 ± 0.9^c7^	34.2 ± 3.3^c7^	34.0 ± 5.0^c7^	33.9 ± 4.0^c7^	33.8 ± 4.5^c7^	33.7 ± 2.2^c7^

*Note*: Mean value (*n* = 3) ± SD on a wet basis; the same superscript letter within the row has no significant difference (*p* > .05), whereas the same superscript within the column has no significant difference (*p* > .05).

Abbreviation: TAE, tannic acid equivalent.

Bold indicates significance was tested at *p* value less than or equal to .05.

**TABLE 4:** Reduction in tannin from blended CAJ using gelatine (mg/100 ml TAE)

Gelatine concentration (g/L)	Time (h)							
	0	1	2	4	6	8	10	12
0	258.0 ± 8.9^a1^	211.0 ± 2.1^b1^	193.0 ± 3.2^c1^	**181.0 ± 1.1** ^**d1**^	180.5 ± 1.0^d1^	180.1 ± 1.8^d1^	179.9 ± 3.6^d1^	179.8 ± 1.7^d1^
0.025	258.0 ± 8.9^a1^	158.0 ± 0.3^b2^	142.6 ± 0.8^c2^	142.2 ± 2.0^c2^	142.0 ± 2.3^c2^	141.9 ± 1.4^c2^	141.8 ± 0.3^c2^	141.7 ± 2.1^c2^
0.05	258.0 ± 8.9^a1^	143.0 ± 1.6^b3^	106.6 ± 4.0^c3^	106.2 ± 1.9^c3^	106.0 ± 4.1^c3^	105.9 ± 0.6^c3^	105.8 ± 2.4^c3^	105.7 ± 2.6^c3^
0.1	258.0 ± 8.9^a1^	138.0 ± 1.9^b4^	78.0 ± 1.8^c4^	77.6 ± 0.6^c4^	77.4 ± 0.8^c4^	77.3 ± 1.9^c2c4^	77.2 ± 1.3^c4^	77.1 ± 2.3^c4^
0.2	258.0 ± 8.9^a1^	129.0 ± 0.8^b5^	55.0 ± 3.7^c5^	54.6 ± 1.5^c5^	54.4 ± 2.8^c5^	54.3 ± 0.9^c5^	54.2 ± 2.9^c5^	54.1 ± 1.4^c5^
0.3	258.0 ± 8.9^a1^	128.0 ± 0.6^b6^	65.0 ± 3.8^c6^	64.6 ± 1.5^c6^	64.4 ± 2.8^c6^	64.3 ± 0.9^c6^	64.2 ± 0.9^c6^	64.1 ± 1.4
0.4	258.0 ± 8.9^a1^	127.0 ± 0.7^b7^	70.1 ± 0.2^c7^	69.7 ± 1.2^c7^	69.5 ± 0.7^c7^	69.4 ± 1.9^c7^	69.3 ± 0.5^c7^	69.2 ± 2.6^c7^

*Note*: Mean value (*n* = 3) ± SD on a wet basis; the same superscript letter within the row has no significant difference (*p* > .05), whereas the same superscript within the column has no significant difference (*p* > .05).

Abbreviation: TAE, tannic acid equivalent.

Bold indicates significance was tested at *p* value less than or equal to .05.

On the other hand, tannin was significantly removed at 2 h of clarification using 0.2 g of gelatine per liter for both pressed and blended juice (*p* < .05). More than 2 h of clarification in both pressed and blended juice did not yield a significant difference in tannin removal (*p* > .05). While a lower concentration of gelatine was inefficient in tannin removal, the use of higher concentration did not yield any further tannin removal in both blended and pressed juice.

Conversely, the unclarified juice presented significant tannin removal (*p* < .05) after 4 h of incubation at ambient temperature. Similarly, further incubation of the juice beyond 4 h did not present any significant further removal of tannin for both pressed and blended CAJ (*p* > .05). Even though tannin in unclarified juice was significantly removed at 4 h, this removal was relatively less compared to that of clarified juice at 2 h. In both blended and pressed juice, tannin removal in clarified juice was more than twofold that of unclarified juice. This implies that, even though tannin reduction can happen naturally without a need for a clarifier, the process is slow and inefficient.

The decrease in tannin contents after the addition of gelatine is explained by the formation of insoluble gelatine–tannin complexes that are deposited at the bottom of the container as flocculates and can be separated from the clear juice through decantation and filtration processes (Naka et al., 2016). Gelatine is a protein that forms a complex with tannin by hydrogen bonding between phenolic hydroxyl group of tannin and carbonyl group of the protein. The interactions are primarily noncovalent governed by the molecular weight, solubility, size, and conformational flexibility of phenolic compounds, proteins, and starch (Naka et al., 2016). The gradual increase in tannin content was observed when higher concentrations of gelatine (beyond 0.2 g/L) were used, which is also a similar trend to Naka et al. (2016). This might be due to the fact that, at higher gelatine concentrations, the tannins remain in the juice in the form of a complex with the gelatine (Prommajak et al., 2018). Therefore, the use of 0.2 g of gelatine per liter of juice was considered optimum for 2 h of clarification at room temperature. After this clarification, the remaining tannin was considered the minimum residual tannin.

### 3.3 Nutritional composition of cashew apple juice

Nutrient contents of blended and pressed clarified and unclarified CAJ are presented herein. The unclarified blended CAJ had high content of: calcium (24.50 mg/100 ml), potassium (265.00 mg/100 ml), phosphorus (2.0 mg/100 ml), zinc (0.80 mg/100 ml), and iron (1.92 mg/100 ml), (*p* < .05) compared to its counterpart, the pressed unclarified CAJ. On the other hand, pressed unclarified CAJ had a significantly higher level of magnesium (40.30 mg/100 ml) (*p* < .05). The higher mineral content in unclarified blended CAJ may be explained by the fact that blending involves crushing the whole fruit including the skin where much of the minerals are concentrated (Czech et al., 2020).

In addition, contents of potassium, calcium, and zinc in unclarified pressed CAJ are higher than those reported in previous studies (Pascal et al., 2018), while iron and magnesium were similar. Conversely, the study showed a significant decrease in minerals after clarification for both pressed and blended CAJ (*p* < .05) (Table 5). The decrease in mineral content following clarification is linked to the precipitation and decantation process where some of these minerals might have been trapped in the precipitates that are wasted.

**TABLE 5:** Minerals, vitamin C, and β‐carotene content of unclarified and clarified CAJ

CAJ (mg/100 ml)	Ca	K	P	Z	Fe	Mg	Vitamin C	β‐Carotene
UPCAJ	19.90 ± 0.2^a^	244.00 ± 4.0^a^	0.8 ± 0.2^a^	0.2 ± 0.13^a^	1.66 ± 0.06^a^	40.30 ± 0.30^a^	289.39 ± 19.49^a^	0.65 ± 0.4^a^
CPCAJ	18.9 ± 2.9^b^	237.16 ± 3.5^b^	0.67 ± 0.0^b^	0.18 ± 0.0^b^	1.56 ± 0.0^b^	37.76 ± 1.9^b^	263.51 ± 9.9^b^	0.62 ± 0.0^b^
UBCAJ	24.50 ± 0.1^c^	265.00 ± 5.0^c^	2.0 ± 0.3^c^	0.80 ± 0.05^c^	1.92 ± 0.2^c^	30.70 ± 0.20^c^	322.94 ± 4.85^c^	1.76 ± 0.13^c^
CBCAJ	22.80 ± 1.4^d^	254.43 ± 4.6^d^	1.6 ± 0.4^d^	0.72 ± 0.1^d^	1.77 ± 0.1^d^	27.94 ± 2.3^d^	290.72 ± 2.3^d^	1.65 ± 0.0^d^

*Note*: Mean value (*n* = 3) ± SD wet basis. The same superscript letter within the row has no significant difference (*p* > .05).

Abbreviations: CBCAJ, clarified blended cashew apple juice; CPCAJ, clarified pressed cashew apple juice; UBCAJ, unclarified blended cashew apple juice; UPCAJ, unclarified pressed cashew apple juice.

The results reveal a significantly higher content of vitamin C in unclarified blended CAJ (322.94 mg/100 ml) than in unclarified pressed CAJ (289.39 mg/100 ml) (*p* < .05) (Table 4). The higher vitamin C in unclarified blended than in pressed CAJ is mainly due to the fact that blended juice contains the skin of the fruit where vitamin C is concentrated (Martí et al., 2009; Lee & Kader, 2000).

In addition, the blended CAJ contains higher phenol which protects vitamin C against oxidative decomposition (Lee & Kader, 2000). This study records higher amount of Vitamin C than other tropical fruits like grape, lemon, mango, orange, and pineapple, which contain an average value of 45.0, 33.7, 30.9, 54.7, and 14.7 mg of vitamin C per 100 ml of juice, respectively (Akinwale, 2000).

Moreover, clarification reduced the amount of Vitamin C significantly for both pressed and blended CAJ (*p* < .05) (Table 5). The oxidation of vitamin C during clarification results in its reduction (Talasila et al., 2012). Vitamin C as ascorbic acid (AA) is easily oxidized in the presence of a metal catalyst, light, oxygen, alkaline pH, and high temperature (Lee & Kader, 2000; Martí et al., 2009). Dihydro ascorbic acid, an oxidized form of AA, can be reversibly reduced to AA and irreversibly oxidized to form diketogulonic acid, which has no vitamin C activity (Lee & Kader, 2000). The incubation of juice at ambient temperature also facilitated the oxidation of vitamin C. Also, the exchange of hydrogen bonding with gelatine and forming a complex facilitated vitamin C reduction (Naka et al., 2016).

The results showed a significant difference between β‐carotene content of the unclarified pressed CAJ (0.65 mg/100 ml) and that of the blended (1.76 mg/100 ml) (*p* < .05) (Table 5). The unclarified blended CAJ had higher β‐carotene content because blending involves crushing the whole fruit where much of the β‐carotene is packed on the skin of the fruit (Rodriguez‐Amaya, 2001). Furthermore, β‐carotene content decreased significantly after clarification (*p* < .05) for both pressed and blended CAJ. This is because β‐carotene exhibits high water insolubility, causing carotene loss during clarification (Karangwa et al., 2010).

Also, β‐carotene is a very reactive compound due to its highly unsaturated structure, consequently prone to isomerization (Pénicaud et al., 2011). Syamila et al. (2019) reported loss of β‐carotene at a low temperature of 20°C. Additionally, β‐carotene undergoes oxidation due to the presence of oxygen in the juice (Pénicaud et al., 2011).

Unclarified blended CAJ had lower sugar content (15,235.0 mg/100 ml) compared to unclarified pressed CAJ (16,078.33 mg/100 ml) (*p* < .05) (Table 6). Blending facilitates the release of an enzyme that causes glycosylation, thereby causing a reduction in the number of carbonyl groups for sugar reaction (Daramola, 2013). Results on total sugar for unclarified blended CAJ concur with those of Msoka et al. (2017), who reported that total sugar value range from 8875.9 to 21,941.4 mg/100 ml. Similarly, lower total sugar content was found in unclarified blended CAJ when compared to the study done by Marc et al. (2012) and Naka et al. (2015). In another study, Marc et al. (2021) reported low total sugar content (8530 mg/100 ml) for unclarified blended CAJ compared to this study.

**TABLE 6:** Antioxidant activity, sugar, and total phenol contents of unclarified and clarified CAJ

CAJ (mg/100 ml)	Total sugar	Sucrose	Glucose	Fructose	Total phenol	Antioxidant activity
UPCAJ	16,078.33 ± 1.67^a^	1110 ± 6.08^a^	5365 ± 55.87^a^	9302.67 ± 50.54^a^	230.37 ± 10.87^a^	78.69 ± 0.26^a^
CPCAJ	15,917.24 ± 1.77^a^	1097.7 ± 3.84^a^	5311.44 ± 9.94^a^	9256.66 ± 2.77^a^	225.88 ± 2.40^a^	76.55 ± 2.35^a^
UBCAJ	15,235.00 ± 2.00^b^	994.67 ± 19.62^b^	5146 ± 21.66^b^	9043.67 ± 9.45^b^	270.37 ± 12.07^b^	99.28 ± 0.43^b^
CBCAJ	15,081.55 ± 10.26^b^	984.66 ± 2.74^b^	5093.52 ± 4.74^b^	8411.88 ± 2.78^b^	265.44 ± 2.62^b^	96.42 ± 1.62^b^

*Note*: Mean value (*n* = 3) ± SD wet basis. The same superscript letter within the row has no significant difference (*p* > .05).

Abbreviations: CBCAJ, clarified blended cashew apple juice; CPCAJ, clarified pressed cashew apple juice; UBCAJ, unclarified blended cashew apple juice; UPCAJ, unclarified pressed cashew apple juice.

Furthermore, unclarified blended juice had lower fructose (9043.67 mg/100 ml) glucose (5146 mg/100 ml) and sucrose (994.67 mg/100 ml) (*p* < .05) compared to unclarified pressed CAJ (Table 6). The results on glucose for unclarified blended CAJ are in agreement with Marc et al. (2012), who reported glucose content of 4720–5680 mg/100 ml; however, the same study reported higher content of fructose (10,070–11,030 mg/100 ml) and lower content of sucrose (250–530 mg/100 ml). Of the types of sugar, fructose is predominant in cashew apples compared to glucose and sucrose (Deenanath et al., 2015; Marc et al., 2012).

Clarification for both blended and pressed CAJ showed a decrease in sugar content but not significant (*p* > .05). The decrease in sugar might be due to the exchange of hydrogen bonding with gelatine (Naka et al., 2016).

Additionally, the total phenolic contents were 230.47 mg GAE/100 ml and 270.37 mg GAE/100 ml for unclarified pressed and blended CAJ, respectively (*p* < .05), Table 6. Even after clarification, blended juice had significantly higher phenolic content (*p* < .05), which is explained by the higher amount of phenol on the fruit skin (Konarska and Domaciuk, 2018; Kyraleou et al., 2019). These results are consistent with the results reported by Msoka et al. (2017) on the unclarified blended CAJ. Phenolic compounds are also considered necessary as they possess antioxidant and anti‐inflammatory properties (Zhang et al., 2011).

Moreover, clarification reduced the phenol content for both blended and pressed CAJ because gelatine has carbonyl group that forms a complex by hydrogen bonding with phenolic hydroxyl groups, hence forming a precipitate that sediments to the bottom of the container by gravity, and are removed through decantation (Prommajak et al., 2019).

Furthermore, antioxidant activity for unclarified blended and pressed CAJ was 99.28% and 78.69% (*p* > .05), respectively (Table 6), whereas after clarification, there was no significant difference (*p* > .05) in antioxidant activity. The high antioxidant content of blended CAJ is due to the high content of ascorbic acid and phenolic compounds (Queiroz et al., 2011).

Following the study by Msoka et al. (2017), who screened the physicochemical and nutritional contents of pressed cashew apple juice across a variety of cashew accession in Tanzania, the current study is the first to report the potential of both blended and pressed CAJ with and without clarification process, their nutrient retention, and antioxidant activities. The technologies can be applied both at household and small‐ to medium‐scale processing; hence, its adoption can contribute not only to the reduction of postharvest losses of CA but also to income generation and food and nutrition security.

### 3.4 Sensory profile of cashew apple juice

**FIGURE 1:**
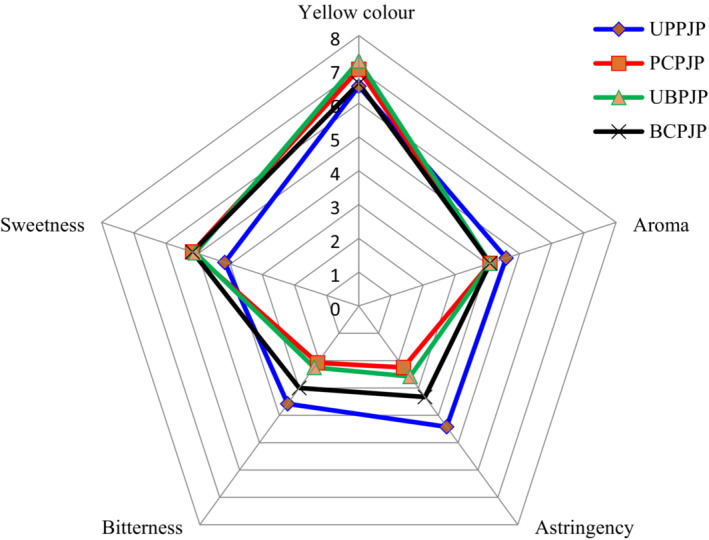
Sensory profile of cashew apple juice. Mean value (*n* = 12) ± SD; BCPJP, blended clarified and pasteurized juice with preservatives; PCPJP, pressed clarified and pasteurized juice with preservatives; UBPJP, unclarified, blended and pasteurized juice with preservatives; UPPJP, unclarified, pressed and pasteurized juice with preservatives

The sensory performance of cashew apple juice is presented in Figure 1. Each sensory descriptor or attribute evaluated is represented by an axis that starts from point 0 (the center) of the scale. The intensity of each descriptor increases toward the edge of the graph. The mean score for each attribute on a particular juice sample is presented in the corresponding axis. The sensory profile of each sample is drawn at the point of connection represented by the mean score for each descriptor (da Silva et al., 2014). Both blended and pressed CAJ received the highest intensity scores for yellow color and sweetness. On the other hand, the lowest intensity of astringency was observed on pressed clarified and pasteurized juice with preservatives (PCPJP).

## 4 CONCLUSIONS

This study demonstrates the effect of different concentrations of gelatine as a clarifying agent in the processing of cashew apple juice. It reveals the potential of using gelatine at low concentrations of 0.2 g/L at a minimum of 2 h of clarification in both pressed and blended CAJ without requiring temperature‐controlled environment. Although there were no significant differences in some physicochemical properties (TSS, TTA, and pH) of unclarified pressed and blended CAJ, the latter presented higher contents of vitamin C, β‐carotene, total phenol minerals, sugar, and antioxidant activities.

The study further revealed a significant decrease in vitamin C, β‐carotene, and minerals following clarification in both pressed and blended juice. Nevertheless, the clarification process retained a substantial amount of key nutrients contributing to food and nutrition security. Besides, sensory evaluation scored high for sweetness and yellow color intensity on both pressed and blended CAJ. Furthermore, future studies to assess the preference of blended and pressed CAJ without clarification in places where astringent taste is not a concern are recommended to explore the potential for additional nutrient retention. Additionally, studies to expound the shelf‐life stability of CAJ are recommended to complement the current effort and provide a basis for potential business investment.

## CONFLICT OF INTEREST

The authors declare no conflict of interest.

## Data Availability

Data are available on request from the corresponding author.
